# Can COVID 19 present like appendicitis?

**DOI:** 10.1016/j.idcr.2020.e00860

**Published:** 2020-06-02

**Authors:** Ahmed Abdalhadi, Mohammed Alkhatib, Ahmad Y. Mismar, Waleed Awouda, Loai Albarqouni

**Affiliations:** a Hamad Medical Corporation, Qatar; b Institute for Evidence-Based Healthcare, Bond University, Gold Coast, Australia

**Keywords:** COVID-19, coronavirus disease-19, WHO, World Health Organization, SARS, severe acute respiratory syndrome, ARDS, acute respiratory distress syndrome, MCV, mean corpuscular volume, CDC, communicable diseases center, ACE2, angiotensin-converting enzyme 2, COVID-19, Appendicitis, Pneumonia, Pandemic, ACE2

## Abstract

Coronavirus disease -19 is a novel pandemic contagious respiratory infection that frequently presents with fever and dry cough. However, it can present with other rare symptoms. As this disease is a new disease, the full picture of the disease presentation is not yet clear, and it might present with symptoms and signs of other common diseases. Here, we report a 40 year old female who presented with acute onset nausea, vomiting, loss of appetite and vague abdominal pain as a clinical picture of appendicitis, but her CT abdomen image showed normal appendix, bilateral patchy peripheral lung basal consolidation, and ground-glass attenuation, so she was tested for coronavirus disease-19, which was positive.

## Introduction

Coronavirus disease-19 (COIVD-19) is caused by severe acute respiratory syndrome Coronavirus 2 (SARS-CoV-2), which started in December 2019 as an epidemic in Wuhan, China [1]. On 11 March 2020, the World Health Organization (WHO) declared it a pandemic after reaching more than 114 countries [2]. Droplet transmission from an infected person to another is the main way of disease transmission. Fever and dry cough are the most frequently reported symptoms in COVID-19 patients. The majority of the COVID-19 patients are mild, but some patients might experience a severe form of the disease with pneumonia, acute respiratory distress syndrome (ARDS), and even death [3]. Previous reports suggested that SARS-CoV-2 can infect the digestive system and presents with gastrointestinal symptoms such as lack of appetite, diarrhea, vomiting, and abdominal pain [4]. However, we are not aware of any reports describe a patient with COVID-19 presented as acute appendicitis. We report a 40-year-old female with COVID-19 patient presented initially with symptoms mimicking acute appendicitis.

## Case report

A 40-year-old woman, married, working as a school housekeeper, presented to the emergency department with a one-day history of abdominal pain. This pain started as a vague, mild generalized pain, then localized to the right iliac fossa and became severe and constant. She denied any change in her bowel motions. It was associated with nausea and recurrent episodes of nonprojectile food content vomiting for 3 days.

She had a febrile sensation, has not been recorded, and a lack of appetite for the last 3 days, but there was no history of nasal congestion, runny nose, sore throat, cough, or chest pain. The patient did not report any contact with sick patients or recent travel to epidemic areas. The patient was previously healthy with no history of chronic medical conditions of relevance. She was afebrile and vitally stable. She had a peripheral oxygen saturation (SpO2) of 100 % on ambient air, a respiratory rate (RR) 17 breath per minute, a heart rate (HR) 72 beats per minute, and blood pressure 123/85 mmHg. The abdomen was not distended and soft, but there was tenderness and rebound tenderness at the right iliac fossa. There was no organomegaly. The examination of the chest and the other systems were normal. Her routine blood examination showed leukopenia with WBC 3000/μL, lymphopenia 800/μL, a microcytic hypochromic anemia with hemoglobin 9.6 g/dl (normal range 12–15), a mean corpuscular volume (MCV) 62.6 fl (normal range 83–101), and platelet level was normal.

**Fig. 1:**
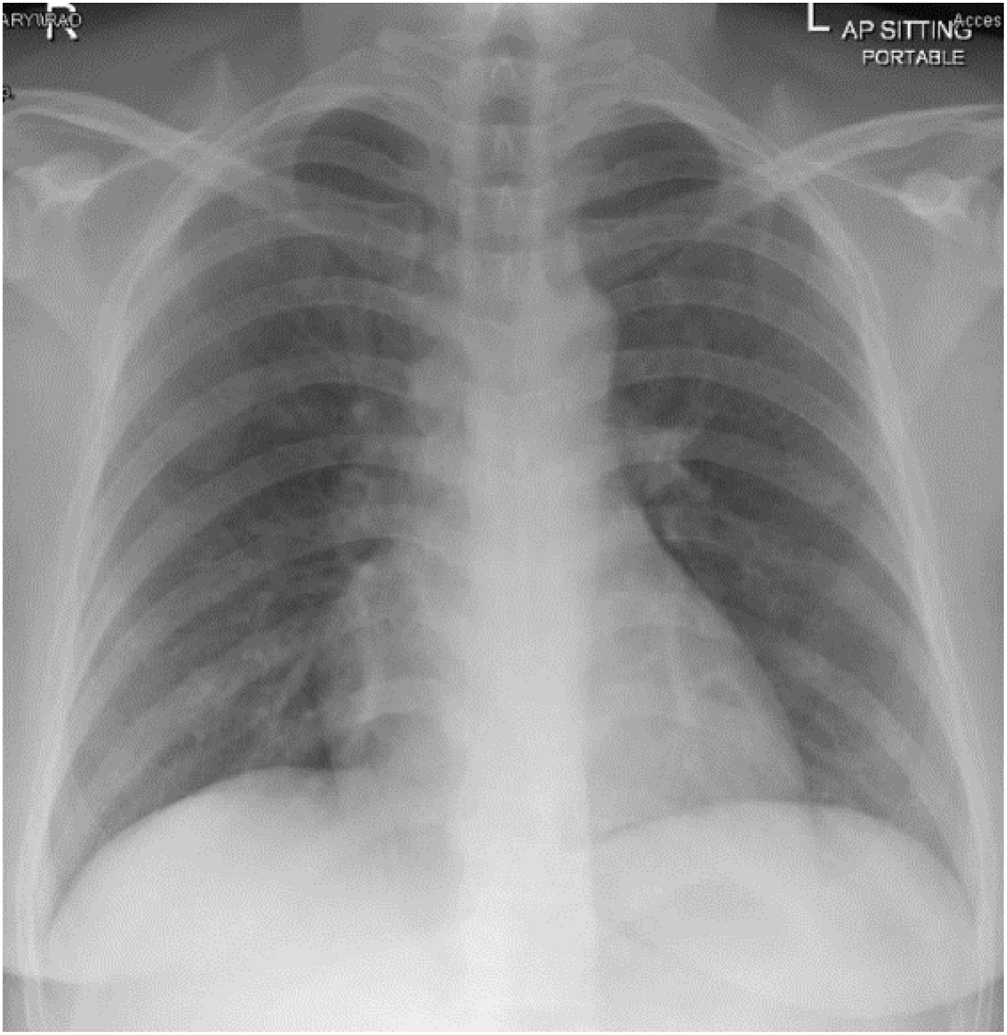
Normal portable AP CXR on the 1st day of admission.

Kidney and liver functions were normal, C-reactive protein 14.4 mg/l (normal range 0–5), and procalcitonin was normal. Pregnancy test was negative. Chest x ray was completely normal initially and after 3 days (Fig. 1).

**Figs. 2 and 3:**
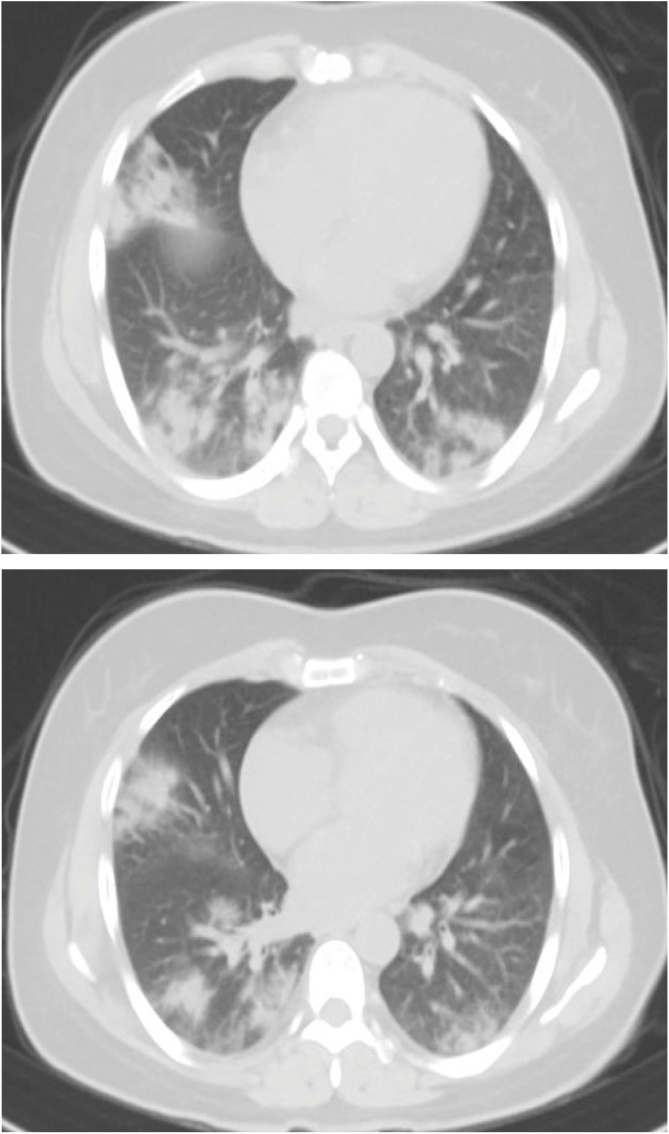
Showed bilateral patchy peripheral lung basal consolidations and ground-glass attenuations.

Based on the history, physical examination and laboratory results, Alvarado score assessment for acute appendicitis was done (5/10) which supported the diagnosis of appendicitis. CT abdomen was ordered to rule out acute appendicitis. The image revealed that the appendix is normal, but there are bilateral patchy peripheral lung basal consolidations and ground-glass attenuations (Figs. 2 and 3 ). Given the current pandemic of COVID-19, the CT scan findings suggested the possibility of COVID-19. Therefore, nasal and throat swabs from the patients were tested for COVID-19. The results of the reverse transcription polymerase chain reaction (RT-PCR) came back positive for SARS-CoV-2.

**Figs. 4 and 5:**
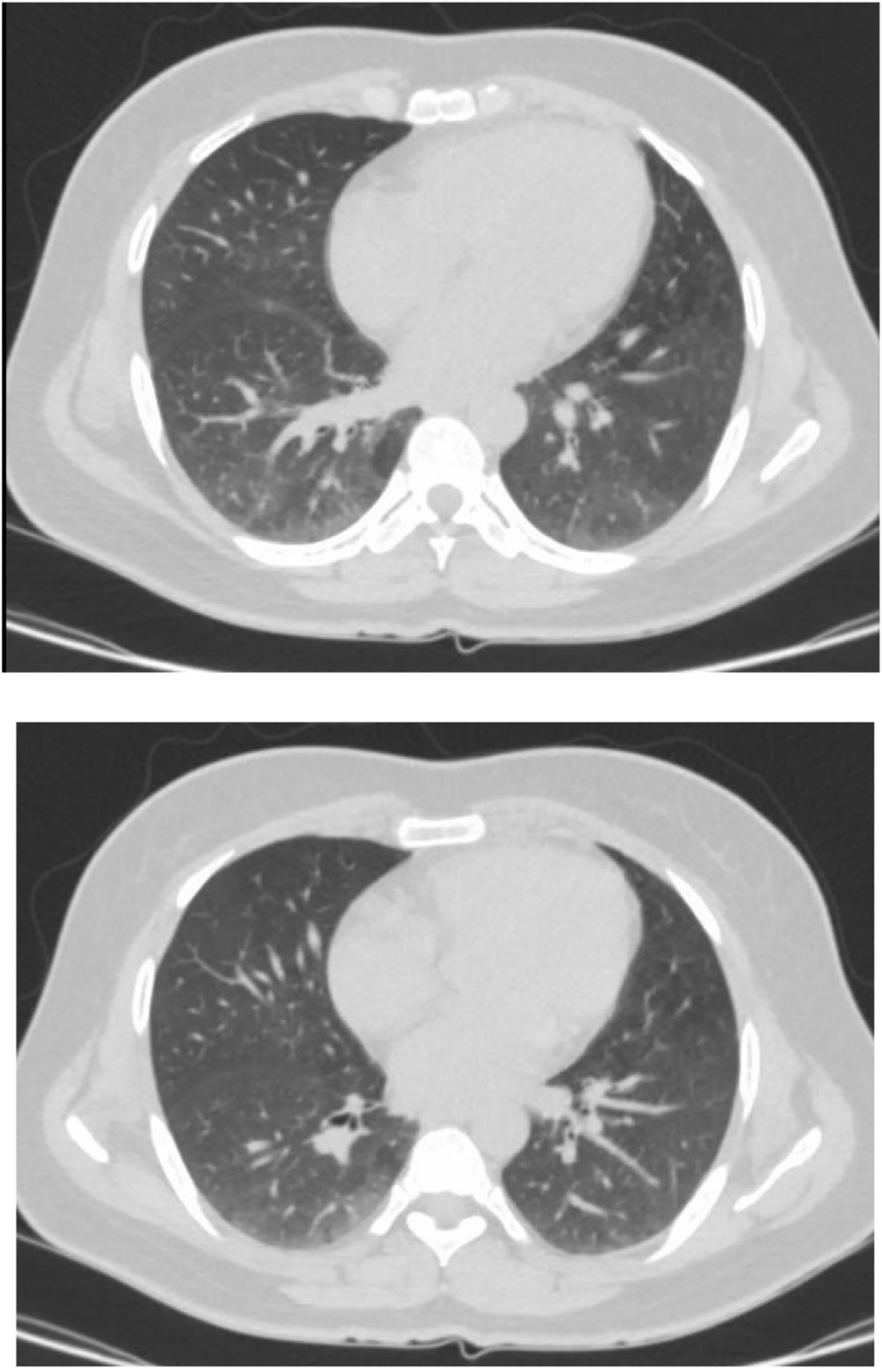
Almost complete resolution of the previously noted patchy lower lung zones consolidation and groundglass opacities (GGO).

She was admitted to communicable diseases center (CDC) under airborne isolation and started on a COVID-19 pneumonia treatment local protocol; azithromycin 500 mg IV daily for 10 days, ceftriaxone 2 g IV daily for 10 days, oseltamivir 150 mg q 12 h for 10 days and darunavir/cobicistat (800/150 mg) for 14 days. She has improved and her symptoms have resolved. The CT chest (image 4 and 5) was repeated after 2 weeks of management to confirm the improvement and it showed almost complete resolution of the previously noted patchy lower lung zones consolidation and ground glass opacities without obvious residual significant ground glass opacity or consolidation could currently be appreciated, however, minimal dependent possible congestive changes still noticed. PCR test was repeated after 2 weeks and still positive (Figs. 4 and 5 ).

## Discussion

SARS-CoV-2, the causative of COVID-19, uses angiotensin-converting enzyme 2 (ACE2), which expressed on the cell membranes of the lungs, intestines, arteries, heart, and kidney cells; as an entry point to get into the cell for replication [5,6]. This explains why this virus could infect intestines and presents with digestive symptoms. Also, it has reported that the virus is shed in the stool, and some patients tested positive with rectal swab real-time PCR (RT-PCR) [7]. Although the main way of the virus transmission among humans is through droplets, the presence of the virus in the stool support the possibility of fecal-oral transmission. So, cleaners, as our patient, are at risk of getting infection from stool [8].

COVID-19 gastrointestinal symptoms are not uncommon. Anorexia, diarrhea, vomiting and abdominal pain are the most common reported symptoms [[9], [10], [11]]. Moreover, these symptoms might appear before the respiratory symptoms [12].

Although, COVID-19 is extremely unlikely to present clinically appendicitis like symptoms (Right lower iliac fossa pain, anorexia, nausea and vomiting), in laboratory and imaging findings, we discovered leukopenia, lymphopenia and ground glass appearance, these findings raised the suspicion of COVID-19 especially the radiological findings in our case (bilateral lung basal consolidations and ground-glass attenuations) were typical for COVID-19 as reported in UK, supporting the value of imaging mainly CT chest in early detection especially in highly suspected patients where CT findings could be positive for viral infection before laboratory results for viral infection [13,14]. These findings were striking and surprising at the same time in era of COVID-19. To the best of our knowledge, this is the third report of a case in the literature of novel COVID-19 was presented initially mimicking appendicitis in clinical history and physical examination [15,16]. Some studies showed that viral infections could cause acute appendicitis in different ways: lymphoid hyperplasia, which leads to appendix obstruction, and mucosal ulcerations, which results in a secondary bacterial infection [17]. However, it is unknown if there is any relation between COVID-19 and acute appendicitis.

## Funding

The publication of this article was funded by the Qatar National Library.

## Contribution

AA, MA, AM conceived and designed the study. AA, AM, WA collected and interpreted the data. AA, MA wrote the Abstract. LA, MA critically revised the Abstract. LA, MA approved the final version of the Abstract for publication.

## Consent

Written consent has been taken from the patient to publish her case and her images.

## Declaration of Competing Interest

The authors report no conflicts of interest in this work.

## References

[1] Chan J.F., Yuan S., Kok K.H. (2020). A familial cluster of pneumonia associated with the 2019 novel coronavirus indicating person-to-person transmission: a study of a family cluster. Lancet.

[2] https://www.who.int/dg/speeches/detail/who-director-general-s-opening-remarks-at-the-media-briefing-on-covid-19---11-march-2020.

[3] Chan J.F., Yuan S., Kok K.H. (2020). A familial cluster of pneumonia associated with the 2019 novel coronavirus indicating person-to-person transmission: a study of a family cluster. Lancet.

[4] Pan L., Mu M., Yang P. (2020). Clinical characteristics of COVID-19 patients with digestive symptoms in Hubei, China: a descriptive, cross-sectional, multicenter study. Am J Gastroenterol.

[5] Zhou P., Yang X.L., Wang X.G. (2020). A pneumonia outbreak associated with a new coronavirus of probable bat origin. Nature.

[6] Hamming I., Timens W., Bulthuis M.L. (2004). Tissue distribution of ACE2 protein, the functional receptor for SARS coronavirus. A first step in understanding SARS pathogenesis. J Pathol.

[7] Xu Y., Li X., Zhu B. (2020). Characteristics of pediatric SARS-CoV-2 infection and potential evidence for persistent fecal viral shedding. Nat Med.

[8] Liu Y. (2020). Aerodynamic characteristics and RNA concentration of SARS-CoV-2 aerosol in Wuhan hospitals during COVID-19 outbreak.

[9] Tian Y., Rong L., Nian W., He Y. (2020). Review article: gastrointestinal features in COVID-19 and the possibility of faecal transmission. Aliment Pharmacol Ther.

[10] Ong J., Young B.E., Ong S. (2020). COVID-19 in gastroenterology: a clinical perspective. Gut.

[11] Jin X., Lian J.S., Hu J.H. (2020). Epidemiological, clinical and virological characteristics of 74 cases of coronavirus-infected disease 2019 (COVID-19) with gastrointestinal symptoms. Gut.

[12] Gu J., Han B., Wang J. (2020). COVID-19: gastrointestinal manifestations and potential fecal-oral transmission. Gastroenterology.

[13] Chua F., Armstrong-James D., Desai S.R. (2020). The role of CT in case ascertainment and management of COVID-19 pneumonia in the UK: insights from high-incidence regions. Lancet Respir Med.

[14] Li Y., Xia L. (2020). Coronavirus disease 2019 (COVID-19): role of chest CT in diagnosis and management. AJR Am J Roentgenol.

[15] Vu D., Ruggiero M., Choi W.S. (2020). Three unsuspected CT diagnoses of COVID-19. Emerg Radiol.

[16] Pautrat K., Chergui N. (2020). SARS-CoV-2 infection may result in appendicular syndrome: Chest CT scan before appendectomy [published online ahead of print, 2020 Apr 15]. J Visc Surg.

[17] Alder A.C., Fomby T.B., Woodward W.A., Haley R.W., Sarosi G., Livingston E.H. (2010). association of viral infection and appendicitis. Arch Surg..

